# “I do not know the advantages of having a general practitioner” - a qualitative study exploring the views of low-acuity emergency patients without a regular general practitioner toward primary care

**DOI:** 10.1186/s12913-024-10977-2

**Published:** 2024-05-15

**Authors:** Lisa Kümpel, Sarah Oslislo, Rebecca Resendiz Cantu, Martin Möckel, Christoph Heintze, Felix Holzinger

**Affiliations:** 1 Charité– Universitätsmedizin Berlin, corporate member of Freie Universität Berlin and Humboldt-Universität zu Berlin, Institute of General Practice, Charitéplatz 1, 10117 Berlin, Germany; 2 Charité– Universitätsmedizin Berlin, corporate member of Freie Universität Berlin and Humboldt-Universität zu Berlin, Division of Emergency Medicine, Campus Virchow Klinikum and Campus Charité Mitte, Berlin, Germany

**Keywords:** Emergency department, GP attachment, GP non-utilization, Continuity of care, Qualitative research, General practitioners, Primary care

## Abstract

**Background:**

Emergency departments (ED) worldwide have to cope with rising patient numbers. Low-acuity consulters who could receive a more suitable treatment in primary care (PC) increase caseloads, and lack of PC attachment has been discussed as a determinant. This qualitative study explores factors that contribute to non-utilization of general practitioner (GP) care among patients with no current attachment to a GP.

**Method:**

Qualitative semi-structured telephone interviews were conducted with 32 low-acuity ED consulters with no self-reported attachment to a GP. Participants were recruited from three EDs in the city center of Berlin, Germany. Data were analyzed by qualitative content analysis.

**Results:**

Interviewed patients reported heterogeneous factors contributing to their PC utilization behavior and underlying views and experiences. Participants most prominently voiced a rare need for medical services, a distinct mobility behavior, and a lack of knowledge about the role of a GP and health care options. Views about and experiences with GP care that contribute to non-utilization were predominantly related to little confidence in GP care, preference for directly consulting medical specialists, and negative experiences with GP care in the past. Contrasting their reported utilization behavior, many interviewees still recognized the advantages of GP care continuity.

**Conclusion:**

Understanding reasons of low-acuity ED patients for GP non-utilization can play an important role in the design and implementation of patient-centered care interventions for PC integration. Increasing GP utilization, continuity of care and health literacy might have positive effects on patient decision-making in acute situations and in turn decrease ED burden.

**Trial registration:**

German Clinical Trials Register: DRKS00023480; date: 2020/11/27.

**Supplementary Information:**

The online version contains supplementary material available at 10.1186/s12913-024-10977-2.

## Background

Many countries record rising numbers of patients in emergency departments (ED) [1, 2]. A substantial share of these ED consulters present with low-acuity care needs and could potentially receive a more suitable treatment in primary care (PC) [3, 4]. Concerning these cases, achieving a shift toward a more appropriate use of general practitioner (GP) care could benefit not only the patients concerned but also the entire ED care system, as crowded EDs are associated with poorer quality of care [5].

The use of ED care for low-acuity complaints is influenced by multiple factors. Among others, a lack of knowledge about alternative acute care options was identified [6, 7] and attributed to a lack of health literacy [8, 9]. There is evidence that lower levels of heath literacy are associated with higher subjectively perceived treatment urgency [9]. In this context, GP care has been advocated not only as an adequate alternative treatment option for low-acuity health issues but also as an important source of health information and facilitator for care coordination, helping patients navigate in the sometimes complex structures of the health care system [10, 11]. Consequently, it is assumed that patients with no continuous GP attachment are more likely to visit the ED for low-acuity care needs [12]. Integrating GP care by reducing access barriers could reduce demand for ED services by both providing a fixed first point of contact and promoting continuity of care, which in turn facilitates the management of long-term health care needs [13–16].

With regard to these findings, it is problematic that every tenth person in Germany has no GP [17]. In our health care system, there is no obligation to register for GP care at a practice and attachment is completely voluntary. Patients are free to choose or change providers anytime, and anyone can likewise visit the ED at own discretion without following any gatekeeping procedure [18]. Having the current absence of regulations and patient steering in mind, it is important to explore the roots of lacking PC integration. This is a prerequisite for the development of concepts to promote attachment and thus to potentially influence future ED utilization behavior [19]. Accordingly, our research questions for this qualitative interview study of ED patients without current attachment to a GP were as follows: What factors are associated with their health care utilization behavior and non-utilization of GP care? Which views and previous experiences of GP care could contribute to GP non-utilization and lack of attachment?

## Methods

### EMAPREPARE study

This qualitative evaluation was conducted as part of the multicenter mixed-methods study EMAPREPARE (Emergency and Acute Medicine– Primary Care Demands in Patients Resorting to Emergency Departments), which is a subproject of the research network EMANet (Emergency and Acute Medicine Network for Health Care Research Berlin). EMAPREPARE explores the redirection potential of low-acuity ED patients without a GP. The project includes a pilot intervention and complementary qualitative interviews. The results and implications of the intervention have been reported in a previous publication [20]. Our paper presents findings on participants’ views and experiences of GP care from the qualitative interview study accompanying the intervention. The core team of researchers for this project consisted of L.K. and S.O. (female health scientists), F.H. and C.H. (male general practitioners and senior researchers), and R.R.C. (female ED physician). The EMAPREPARE study was registered a priori in the German Clinical Trials Register (trial registration number: DRKS00023480, registration date 27/11/2020) [21]. Qualitative data are reported in this article according to the COREQ guidelines (Additional file 2) [22].

### Participants

Interviewees were selected as a subsample of the EMAPREPARE cohort. Recruitment was conducted in three inner city EDs in Berlin, including two university medical centers. Patients were recruited in the ED waiting room, informed about the project’s aims, and asked for written informed consent to participate in the study and an optional qualitative interview. Consent included audio recording, storage and pseudonymized analysis of the interview material. The EMAPREPARE inclusion criteria needed participants to be of age (≥ 18 years) and fluent in German. Patients also had to be self-referred walk-in outpatient cases, triaged in the Manchester Triage System (MTS) lower priority categories 3–5 [23]. A further eligibility prerequisite was that patients did not have a regular GP, meaning no current self-reported attachment to a particular practice, or regular recent visits. Patients were excluded if they were not able to formally give informed consent. Researchers had no relationship with the participants prior to the study. The patients’ reasons for refusing to participate in the study were not recorded.

Recruitment for the interviews was based on the principle of purposive sampling [24]. We tried to balance gender and intentionally over-represented participants willing to make use of the GP appointment service, which was part of the pilot intervention. Further information on sampling details can be found in a previous paper [20]. Patients who consented to a qualitative interview were called after ED discharge to schedule an interview appointment within two weeks after ED presentation to facilitate recall.

### Data collection

A semi-structured interview guide based on the literature was compiled with the intention to capture and understand patients’ views and experiences [25]. The questions were thoroughly discussed within the study team and in an interdisciplinary methods working group at the institute, and pilot tested in two interviews. After the first two interviews, the guide was revised further. The final interview guide consisted of three parts (see Additional file 1 for complete interview guide). The results presented here correspond to the first part of the interview guide, which addressed patients’ experiences and views about GP care (see Table 1), while parts two and three covered participants’ views on the EMAPREPARE intervention, with results reported elsewhere [20]. During the interviews, questions were individually adapted to the conversation flow.

**Table 1:** Excerpt of the interview guide’s questions

Factors for non-utilization, experiences and views about GP care
• Who do you usually turn to when you are ill?
• Please tell me why a GP does not play a role in your health care.
• What experiences have you had with GPs?
• What does it mean for you to “have a regular GP”?

Interviews were carried out between March 2021 and January 2022 by L.K. and S.O. by telephone (to avoid COVID-19 infection risk) and were concluded after thirty-two interviews once no more new themes emerged, indicating content saturation [26]. The interviewees did not receive any questions in advance, and interviews were conducted only once with each patient. Interviews were audio-recorded, and field notes were taken to document additional impressions or specifics of interview circumstances. Recordings were then transcribed verbatim and pseudonymized by L.K. and S.O., the transcripts were not returned to the patients for corrections and/or comments.

### Data analysis

The transcripts of the interviews were analyzed by qualitative content analysis according to Mayring using a combined deductive and inductive approach for coding text segments. This interpretative but rule-guided process is based on coding guidelines. The method allows focusing on essential interview topics and facilitates thematic structuring and summarizing of the content [27]. Due to the exploratory nature of the study, no pre-existing framework was used to guide analysis. The first draft of the coding tree was based on the themes of the interview guide, and additional themes were then inductively derived from the material during coding. This allowed for consideration of both theoretical aspects and interview content. All derived codes had defined coding rules and anchor examples. Transcription, coding, and data analysis were performed in MAXQDA 2020. The majority of interviews were independently coded by two scientists (L.K. and S.O., experienced in qualitative research) to enable comparison and discussion of discrepancies. As interviews were conducted in German, quotes were translated to English by the authors for presentation in this paper.

## Results

### Sample characteristics

Thirty-two interviews were conducted. An overview of the participants’ characteristics is given in Table 2. The interviews had a mean duration of 20 min.

**Table 2:** Characteristics of interviewees (*n* = 32*)*

		*n (%)*
Gender	Female	15 (46.9)
	Male	17 (53.1)
Age groups (years)	20–29	10 (31.2)
	30–39	15 (46.9)
	≥ 40	7 (21.9)
	mean	32.9
	median	32
	min./max.	20/52
Migration history^a^	First generation	10 (31.3)
Previous ED visits in the past six months^b^	Patients with visit mean median min./max.	8 1.25 1.0 1/3
Previous GP visits in the past six months^c^	Patients with visit mean median min./max.	11 1.36 1.0 1/3

^a^Participant not born in Germany; determined post-hoc from the EMAPREPARE quantitative dataset (no sampling criterion)

^b^At least one visit in the past six months (excluding the current visit), mean and median refer to patients with at least one visit

^c^At least one visit in the past six months, mean and median refer to patients with at least one visit

After categorizing the interview data, diverse factors contributing to the non-utilization of GP care emerged. These factors were in turn associated with thematic categories related to patient characteristics and underlying views and experiences with past GP care (see Table 3). In the following, these thematic categories are presented in detail with exemplary interview quotes.

**Table 3:** Factors contributing to non-utilization of GP care: Thematic categories and subcategories

Thematic categories	Subcategories
Patient characteristics related to non-utilization of GP care	• Rare need for medical care • Distinct mobility behavior • Lack of knowledge about the role of a GP and health care options
Views about and experiences with previous GP care	• Little confidence in GP care • Preference of consulting specialists • Negative experiences with GP care
Views on the concept of having a regular GP	• Potential benefits of care continuity

### Patient characteristics related to non-utilization of GP care

#### Rare need for medical care

With regard to the reasons for the low relevance of GP care for their individual care context, interviewees most prominently described a rare need for regular medical care in the past due to their good state of health.

“There is no doctor I have consulted more than five times, except for my gynecologist.” (P23).

“So, I have not really been ill until now and therefore I do not have a GP I regularly visit.” (P20).

#### Distinct mobility behavior

Apart from the rare need for regular medical care, many patients attributed the lack of continuity in their GP utilization patterns to attachment difficulties rooted in their individual mobility patterns.

“Before I came to Germany, I had a GP, but since I moved, I no longer have a GP.” (P28).

“I have simply moved too often. Quite often within Berlin, in different cities, abroad.” (P1).

“My parents still have the same GP that I had as a child. That has changed in modern times because people are much more mobile and move around more often. You no longer stay in one place for twenty to thirty years and have all your doctors in the neighborhood for your whole life.” (P18).

Due to circumstances such as the aforementioned infrequent need for medical care and distinct mobility behavior, many participants depicted a rather situational consultation pattern with sporadic visits to various physicians based on short-term needs. In this context, a personal relationship and continuous attachment to a specific GP practice were frequently described as less relevant than solving acute health problems by consulting a doctor selected on an as-needed basis.

“There is a GP practice where I go when something comes up. However, I do not consider this my GP of choice.” (P26).

“Otherwise, I just do not have any relationship [to a GP] at all because I always just sat down in the acute consultation of some doctor. I described the problem and was treated once-off.” (P25).

“As I said, I do not really have a GP in the true sense. I have been to GPs here and there, depending on which district I was living in at the time, and whether it was an urgent matter or not” (P18).

#### Lack of knowledge about the role of a GP and health care options

Many of the interviewed patients reported a self-perceived lack of knowledge about the responsibility of a GP.

“I do not truly understand [what a GP does], I am of course familiar with the word though.” (P1).

“I do not know the advantages of having a GP. That is why I never truly thought about it.” (P26).

“It is probably good to have an overview of the types of occasions for which people go to the GP. […] because it is not at all clear to me, actually.” (P11).

Some interviewees explicitly mentioned this lack of knowledge about GP care as a factor that made it difficult for them to navigate the care system and find the right doctor for their specific health problems.

“Then it is difficult for me to say, if I have an issue with my ears, whether I should go straight to an ear, nose and throat specialist, or whether I should go to the GP first.” (P13).

“And it is not so clear to me now to what extent the tasks of the GP overlap or differ from the tasks of the respective specialists to whom I have turned thus far. If I were aware of what a GP actually does differently and how this could be of use to me, then I naturally would be open to it.” (P2).

In this context, one patient with a migration history depicted his limited understanding of German health care structures and the role of the GP within the system.

“Because I cannot understand the health system in Germany. It is very different from my country and completely ineffective. In my country you automatically have a GP. Everyone has.” (P28).

### Views about and experiences with previous GP care

#### Little confidence in GP care

Several interviewees also attributed their low utilization of GP care to a lack of confidence in the skills and knowledge of GPs compared to other specialists.

“I always have such a bad feeling about GPs, so sorry about that.” (P4).

“From my experience, it is always the case that the normal doctors [GPs] are a little less experienced and can help a little less with acute cases. They can give great check-ups, they can give great recommendations […], little things like that. Unfortunately, this is not the case for acute cases. They have no experience.” (P4).

“I also understand that the GPs are often not extremely qualified here […], the specialists are usually much more qualified and I do not expect anything.” (P16).

Some patients also considered consulting a GP time-consuming, complicated, and a pointless additional step in the care process. In this regard, patients portrayed the GP as a mere intermediary to medical specialists.

“My general experience is that going through GPs just delays everything even more. However, that is probably a perspective you have as a young person.” (P11).

“I […] find it cumbersome to be sent from a GP to a specialist. It is an outdated concept for me.” (P25).

#### Preference of consulting specialists

Consequently, direct consultation with medical specialists was a pattern of utilization frequently depicted in the interviews.

“When I look back, whenever I went to the doctor, it was usually directly to specialists.” (P12).

“If I am concerned about a specific problem anyway, then I can also sit down in the emergency consultation hour of the specialist.” (P2).

In addition to the aforementioned view that GP care is an intermediary step to be bypassed, some patients explicitly expressed a belief in the professional superiority of medical specialists over GPs.

“Because I often have the feeling that when I go to the GP, he does not really know what to do either, and that I always end up with a specialist.” (P7).

#### Negative experiences with GP care

Regarding past contacts with GP care and their potential role in explaining current individual consultation decisions, some respondents described negative experiences. An important theme in this context was frustration about long waiting times for appointments and in practice.

“ […] I have also always experienced GP surgeries as very crowded.” (P3).

“You cannot always get to an acute consultation right away. You also have to wait.” (P25).

In addition, many participants described previous access problems, such as not being able to obtain a timely GP appointment, or futile attempts to find a practice that would accept new patients.

“I felt very rejected. I called different doctors’ offices and they said, “Do not come!” (P15).

“For three or four months I was looking for a GP, but the answer is always that they do not take new patients.” (P29).

Participants also reported that they had not been satisfied with the treatment they received in the past from the GP.

“When I think about my GP experience, they were less able to help me there.” (P21).

“Thus far, my experience has not been so good, which is why I went straight to the ED. When I had truly severe pain, they [GPs] only ever prescribed me painkillers.” (P27).

Some interviewees described experiencing GP care as impersonal, including the impression that the respective GP was overworked and did not take enough time for consultations.

“[…] because the GPs are so overburdened.” (P16).

“That is always so sobering, you ask yourself, has he [GP] truly listened to what you have to say? You tell him and he types on the computer and you get a prescription and that is it. This personal factor is also missing. It does not exist like that anymore.” (P19).

### Views on the concept of having a regular GP

Participants interviewed were also asked what the concept of ‘having a regular GP’ implied for them. While most patients had a general idea of this, for some it seemed to be a completely foreign concept.

“So probably, it [having a regular GP] just means that someone has a regular doctor that they always go to.” (P1).

“I do not know [what it means to “have a regular GP”].” (P23).

“I do not truly understand it, the term is familiar to me, of course, and I have observed with my grandmother, for example, that she had something like a GP who actually also came to her home. In addition, she knew him for decades.” (P1).

Although some patients did not understand the concept or need for a GP, most interviewees indicated that they could see clear benefits from the continuity of GP-based care. Having a fixed contact person in case of illness who knows the individual medical history was frequently mentioned as the main advantage.

“To have a doctor where you can go if something is wrong and who also knows you and already has the data.” (P30).

“I would say someone who actually knows me. Someone I do not just go to when I have cut off my finger, but who actually knows my history and accompanies me like that. Maybe not through life, but at least for a period of life. Who can then perhaps also assess what the better treatment options are, because I have certain previous illnesses, or because they know that I take certain other medicines or have taken certain other medicines until recently?” (P6).

## Discussion

### Summary of findings

In the interviewed sample of low-acuity ED patients without a regular GP, a number of central contributing factors for GP non-utilization and an associated lack of continuity of care were identified. Patients’ characteristics and lifestyles are linked to underlying views about PC and individual past experiences. In particular, a rare need for medical care due to good general health, mobility, and a lack of knowledge about the role and responsibilities of GPs and health care options were identified as important factors for GP non-utilization. Little confidence in PC providers emerged as a widespread view, possibly contributing to a preference for specialist care. Interviews suggested that this constellation is often due to negative experiences with GP care in the past.

### Results in context

#### Implications of sample composition and study setting

Our study investigated factors that contribute to GP non-utilization in low-acuity ED patients. With regard to our interview sample, it is important to note that it consisted of relatively young patients, corresponding to the overall mean age of 30.6 years in the EMAPREPARE cohort. Regarding the prevalence of a first-generation migration history (∼ 30%), the qualitative sample likewise reflects the composition of the larger cohort from which it was recruited. The two mentioned sample characteristics have been identified in previous studies as factors that increase the odds of not having a GP [17]. Furthermore, previous work has described young age and not having a GP as factors contributing to low-acuity ED usage, which is also consistent with our findings [28, 29]. However, other population groups have also been identified as contributive to rising ED utilization, particularly older people and people living in nursing homes, which is not reflected by our results as to the selection criteria of the study [30].

The results might also reflect specifics of the urban study setting with a high availability of specialists and care choices, where patients are less tied to one provider and have many options, which may play a role in GP care utilization and attachment motives. Other studies also found that low-acuity ED patients in urban settings, compared to rural settings, are less connected to GP care [31] and show lower commitment to their PC provider [11].

#### Understanding utilization motives and potential implications

##### Need to adapt GP care to individual life situations and to diminish access barriers

Many of the comparably young patients from our cohort reported being rarely ill and having no regular need for medical care. Concerning age, findings by Tillmann et al. show that young people are often attached to a pediatrician during their childhood and might miss the transition to GP care as young adults. The authors stress the importance of improving this transition to support GP attachment [17].

Even though utilization of GP care was depicted as rare and sporadic in many interviews, some of the participants notably consulted a GP in the past 6 months (see Table 2). This utilization was prevailingly described as situational, with no attachment or long-term continuity. Accordingly, a qualitative study on the attachment of patients to GP practices by Frederiksen et al. highlighted that patients with higher morbidity and vulnerability have a greater need to have a regular GP [32]. This is consistent with our findings.

Against the backdrop of patient characteristics associated with non-utilization, such as young age, good health and related situational contacts with GP care, it is worth taking a closer look at negative experiences with PC depicted by the participants. Access problems (waiting times, appointment scheduling problems) play an important role in this context. Difficulties in obtaining a timely appointment at a GP practice were also described in a qualitative study from France by Durand et al. and identified as a reason for seeking low-acuity ED care [33]. Access problems may also be related to the problem of patient mobility, which in turn is a feature associated with younger age groups. For people who move between neighborhoods or cities, it might be quite burdensome to connect to a GP and to schedule appointments. The interviews describe experiences of rejection by practices, even for acute complaints. In a recent qualitative study, Korczak et al. investigated determinants of low-acuity ED presentations and found three main factors specifically associated with GP care: having no GP, failure to attend an appointment, and negative previous experiences with a GP practice. In turn, the main reason for not having a GP identified by this study was that patients move around or do not understand the health care system and the most appropriate care paths. The authors suggested that there should be services to enable patients to find a GP who meets their individual needs, arguing that this would increase GP attachment and continuity of care in the long term [34]. A targeted GP attachment program, as piloted in the interventional module of EMAPREPARE, could help to connect patients with GPs [20].

Another aspect underlying the deliberate non-utilization of GP care, which came up repeatedly in our interviews, was little confidence in GP care, which was related to negative experiences with PC in the past. In an Australian study, Wong and Hall examined how ED patients’ experiences with GP care affected their ED attendance and found that patients who had negative experiences with GP care in the past were more likely to visit the ED [35]. A general lack of trust in GP treatment can also play a role in ED utilization decisions [31]. Previous research by our study group has also stressed the potentially negative influence of GP-aversive views and negative PC experiences on utilization behavior [36].

##### Need for patient education

Patients often do not know where to access appropriate care for their needs, or struggle to obtain suitable medical attention. A surprising finding in our data was that many interviewed patients were not familiar with the role and tasks of a GP and the concept of having a regular GP practice. Even if these statements cannot be generalized – especially regarding other settings and populations – they could indicate a lack of health literacy in this patient group. While we did not survey health literacy in our study population, other works however have shown lower health literacy in low-acuity ED patients than in the general population [37, 38]. On that score, Strauß et al. found that health literacy in low-acuity ED patients was positively related to GP attachment. Therefore, they assumed that improving GP attachment among these patients would help them to receive personalized information from their GP about different health care options and therefore make more adequate ED utilization decisions [10]. Likewise, Oedekoven et al. stressed the importance of GPs as a source of health-related information [39].

In line with the theme that non-utilization of GP care is associated with knowledge deficits about the functions and potential of PC, our interviews indicated that beliefs about the professional superiority of specialists frequently seem to play a role in not having a regular GP. Promoting information about the role of GPs and their care capabilities seems crucial in this context. However, it is certainly a challenge to reach people who have no contact with PC. Himmel et al. suggested that– especially for younger patients who are frequently not attached to a GP– health insurance personnel could educate patients about the benefits of having a GP and continuity of care [40]. Other authors have likewise stressed that specific information on the importance of having a regular GP could help to increase patients’ commitment to PC [11]. For patients with utilization patterns that are detached from PC, the ED may appear to be a particularly attractive care pathway or may be perceived as without alternatives. A patient-oriented approach to promoting and improving health literacy is therefore important to support informed decision-making processes [9]. Altogether, we must stress that our results highlight the need to improve the public perception of GPs’ important coordinating role in the healthcare system as well as their medical expertise.

#### Attachment to a GP and continuity of care

Interestingly, our interviews revealed that many patients embrace the theoretical concept of having a regular GP and the associated advantages of having a health care provider who knows their medical history and is available as a point of contact for any medical problems that arise. However, translating this agreement in principle into actual utilization reality is not achieved, with barriers mentioned in our interviews likely playing a central role.

Numerous works have emphasized the link between continuity of care and potentially inadequate ED utilization, and promoting PC attachment appears to be a promising leverage point for streamlining utilization [41–43]. A study by van den Berg et al. showed that patients with regular GP attachment are more likely to consider PC as a primary care option in an acute situation [12]. Other works have stressed that in addition to continuity of care, the doctor-patient relationship plays a central role in the decision to either consult a GP or turn to the ED for a problem that is perceived as urgent [41, 42]. In line with this, Strauß et al. reported that both the quality of a GP-patient relationship and the experienced continuity of care are crucial factors in reducing ED utilization beyond mere attachment to a GP [10]. Our own research points in a similar direction [20]. Notably, the importance of continuity of care extends well beyond the acute care context, and some studies even suggest potential benefits in terms of mortality [42, 44, 45].

Our findings of a fundamental openness to attach to primary care in this patient group however raise the question of how this can best be promoted. The study’s approach is based on voluntary participation in an appointment scheduling service, but this is by no means the only conceivable measure. Internationally, many health care systems (e.g. Denmark) are based on mandatory registration with a particular practice, frequently associated with gatekeeping regulations [46]. Respective policy changes toward a primary care-based healthcare system could alleviate many of the issues raised in our study, with our findings suggesting that this could potentially be well accepted. However, as this was not part of our research question, further investigation is required.

### Strengths and limitations

This qualitative study provides new insights into the views and experiences of low-acuity ED patients without PC integration toward GP care, and their complex reasons for non-utilization. While conducting our study, measures such as independent coding and reflection of results with independent researchers were taken to reduce interviewer bias, but such cannot be completely eliminated [47, 48]. Other caveats include possible bias due to social desirability among interview respondents [49] and selection effects related to patients who may have felt offended when approached by a project about redirection to a GP and the appropriateness of their visit, and therefore refused to participate in the study. A member check with interviewed patients was not performed. Furthermore, qualitative research is inherently subjective, and characteristics of the sample must be considered when reflecting on the results [50]. Due to the inclusion criteria of the pilot study, our qualitative results only reflect the views of a selected population of patients without GP attachment. However, for context and potential contrast with a less selective patient sample, we can refer to a previous qualitative study by our research group [36]. The fact that only patients who had sufficient German language skills were able to take part in the study may also limit transferability to unselected ED users. Moreover, it must be stressed that GP attachment is not formalized (and therefore not associated with e.g. a registration process) in Germany, and ‘having a GP’ or not is a personal subjective definition for the individual patient. As described, part of our sample had made use of GP care in the recent past, but nevertheless participants described themselves as ‘unattached’ to a GP. Apart from the fact that our results reflect peculiarities of the German health care system with its absence of gatekeeping regulations, the urban study setting, as mentioned above, might also have distinctly impacted the results and limits the conclusions derived from it. This requires further research to correspondingly explore the issue in rural settings, for example.

## Conclusions

Factors that contribute to non-utilization of GP care in low-acuity ED patients are multifaceted. Patients’ personal life situations, health literacy, and experiences with GPs, play an important role in their behavior when seeking medical care and choosing acute care options. As the themes identified were found to be interrelated, this study highlights individual non-utilization as a complex configuration, for which the insights derived from our data provide a framework useful for understanding and better description. This can be very helpful in targeting future intervention approaches to promote PC attachment and continuity of care and in turn strengthen GP-mediated health literacy. Our EMAPREPARE pilot intervention is a first step in this direction, providing both information material about alternative care paths and an optional GP appointment scheduling service [20].

### Electronic supplementary material

Below is the link to the electronic supplementary material.

Supplementary Material 1

Supplementary Material 2

## Data Availability

The datasets used and analyzed in this study are available from the corresponding author upon reasonable request.

## Abbreviations

ED: emergency department
PC: primary care
GP: general practitioner
EMANet: Emergency and Acute Medicine Network for Health Care Research Berlin
EMAPREPARE: Emergency and Acute Medicine– Primary Care Demands in Patients Resorting to Emergency Departments
MTS: Manchester Triage System
