# Supplementary material for: “I do not know the advantages of having a general practitioner” - a qualitative study exploring the views of low-acuity emergency patients without a regular general practitioner toward primary care
Source: BMC Health Serv Res. 2024 May 15;24:629. doi: 10.1186/s12913-024-10977-2 (PMC11097521; doi:10.1186/s12913-024-10977-2)
Supplement: Supplementary file 1 — Supplementary Material 1 [file 12913_2024_10977_MOESM1_ESM.docx]

| **Additional file 1: Interview guide for patient interviews in the EMAPREPARE study** | |
| --- | --- |
| **Introductory Question** | |
| - Please tell me how your current emergency department (ED) visit came about. | |
| - What was the deciding factor for you to visit the ED? | |
| **Part 1** | |
| **Factors for non-utilization, experiences and views about GP care** | |
| - Please tell me if a general practitioner (GP) played a role in your decision to visit the ED and why. | |
| - What other people played a role in your decision to visit the ED? | |
| - Who do you usually turn to when you are ill?^1^ | |
| - Please tell me why a GP does not play a role in your health care.^1^ | |
| - What experiences have you had with GPs?^1^ | |
| - What does it mean for you to "have a regular GP"?^1^ | |
| **Part 2** | |
| **Information material** | |
| - How did you feel about receiving an information leaflet about ED alternatives?^2^ | |
| - What did you like or dislike about the information material?^2^ | |
| - What additional information would you have liked to see included in the information material^2^ | |
| - Where do you see a need for change in the information material?^2^ | |
| - Where would you like to find such information material?^2^ | |
| **Part 3** | |
| **Optional GP appointment scheduling service** | |
| **Patients who made use of the service** | **Patients who did NOT make use of the service** |
| - At the time of you ED visit, we did arrange a general GP appointment for you. What is your opinion about this service?^2^ | - Why were you not interested in having a GP appointment arranged^2^ |
| - What were/are* your expectations regarding this GP appointment?^2^ |  |
| - What was/is* particularly important to you regarding your GP appointment?^2^ |  |
| - Could you imagine connecting to this (or any) GP in the long term?*^,2^ - Under which circumstances would you stay with this (or any) GP?*^,2^ | - If you imagine being ill again at some time in the future, to what extend would GP care be an option for you?^2^ |
| - On what occasions could you imagine visiting a GP in the future?^2^ | - On what occasions could you imagine visiting a GP in the future?^2^ |
| **Closing question** | |
| - Can you think of anything else to add to the issues raised, or is there anything that has not been addressed yet? | |

*Phrasing dependent on whether the appointment had already happened.

^1^ Interview guide questions related to the findings reported in this paper (Part 1)

^2^ Interview guide questions related to the findings reported in: Kümpel L, Oslislo S, Resendiz Cantu R, et al. Exploring the views of low-acuity emergency department consulters on an educational intervention and general practitioner appointment service: a qualitative study in Berlin, Germany. BMJ Open 2023;13:e070054. doi:10.1136/bmjopen-2022-070054. (Part 2 and 3)
